# Somatosensory processing in deaf and deafblind individuals: How does the brain adapt as a function of sensory and linguistic experience? A critical review

**DOI:** 10.3389/fpsyg.2022.938842

**Published:** 2022-10-17

**Authors:** Agnes Villwock, Konstantin Grin

**Affiliations:** Sign Languages, Department of Rehabilitation Sciences, Humboldt-Universität zu Berlin, Berlin, Germany

**Keywords:** Somatosensory processing, deafness, deafblindness, signed languages, tactile languages, linguistics, neuroplasticity

## Abstract

How do deaf and deafblind individuals process touch? This question offers a unique model to understand the prospects and constraints of neural plasticity. Our brain constantly receives and processes signals from the environment and combines them into the most reliable information content. The nervous system adapts its functional and structural organization according to the input, and perceptual processing develops as a function of individual experience. However, there are still many unresolved questions regarding the deciding factors for these changes in deaf and deafblind individuals, and so far, findings are not consistent. To date, most studies have not taken the sensory and linguistic experiences of the included participants into account. As a result, the impact of sensory deprivation vs. language experience on somatosensory processing remains inconclusive. Even less is known about the impact of deafblindness on brain development. The resulting neural adaptations could be even more substantial, but no clear patterns have yet been identified. How do deafblind individuals process sensory input? Studies on deafblindness have mostly focused on single cases or groups of late-blind individuals. Importantly, the language backgrounds of deafblind communities are highly variable and include the usage of tactile languages. So far, this kind of linguistic experience and its consequences have not been considered in studies on basic perceptual functions. Here, we will provide a critical review of the literature, aiming at identifying determinants for neuroplasticity and gaps in our current knowledge of somatosensory processing in deaf and deafblind individuals.

## Introduction

Our brain constantly receives and processes signals from the environment and combines them into a multisensory percept—resulting in the most reliable information content. The perceptual system is not fully present at birth but develops as a function of individual experience, which thus shapes brain functions (Bavelier and Neville, 2002; Knudsen, 2004). The different senses are specialized for different stimulus features. While vision offers the most precise information for spatial perception, audition is the most dependable channel for temporal information, and touch for texture perception (Welch and Warren, 1980).

In the case of sensory deprivation, the nervous system changes as a function of the altered input. A total sensory deprivation from birth—such as congenital deafness or deafblindness—can be a unique model for expanding the knowledge about the prospects and limitations of neuroplasticity (for reviews see Merabet and Pascual-Leone, 2010; Pavani and Röder, 2012). However, to date, the network and interplay of the different sensory modalities have not been fully understood. How does the brain respond to unisensory (deafness) or bisensory (deafblindness) deprivation? What is the impact of the age of onset? To which degree might language experience and age of language exposure impact basic perceptual functions, and does language modality matter? All these questions can be linked to the broader context of neuroplasticity. So far, the answers have remained inconclusive.

Neuroplasticity is defined as the ability of the brain to adapt its organization to the specific sensory experiences of an individual (Sheedlo and Turner, 1992). Changes can be observed on the structural level, such as alterations of axonal, dendritic, and synaptic morphologies, or a functional level, that is, modulations in the weights of synaptic connections (Knudsen, 2004). Although the brain remains plastic throughout the lifespan, adult plasticity is more limited in both qualitative and quantitative aspects as compared to developmental plasticity (Knudsen, 2004). Neuroplasticity after sensory deprivation can be classified into two types, corresponding to the brain area in which the change occurs (for reviews see Pavani and Röder, 2012; Heimler et al., 2014). *Intramodal plasticity* refers to changes in a brain area that is typically associated with the processing of a spared modality, such as higher cortical volume in visual areas of deaf participants in comparison to hearing individuals (Bottari et al., 2011; Allen et al., 2013; Scott et al., 2014). For example, Allen et al. (2013) found a larger volume of gray matter in primary visual cortex of congenitally deaf individuals than in both hearing signers and non-signers. This review will focus on studies on *crossmodal plasticity*, which concerns changes in areas that are typically associated with a modality that is not received and processed. Examples are higher activity in auditory areas in deaf compared to hearing individuals during visual stimulation (Finney et al., 2001; Fine et al., 2005; Benetti et al., 2021) and tactile stimulation (Karns et al., 2012; Zimmermann et al., 2021), or activity in visual areas of blind individuals who are listening to speech (Bedny et al., 2011). Benetti et al. (2021) presented patterns of moving dots and observed significantly stronger activation in typical auditory areas in early deaf participants compared to hearing signers and non-signers. Moreover, activation of auditory areas in early deaf signers has been observed during sign language processing (Nishimura et al., 1999; Petitto et al., 2000). These findings point to the relevance of visual motion and visual language input for studies on neuroplasticity in deaf individuals. Importantly, crossmodal reorganization does not follow a random pattern but seems to be functionally selective. As a result, brain regions sustain their typical function in deprived individuals but process it in a spared sense instead (Dormal and Collignon, 2011). Indications of functional selectivity have been shown in studies on visual processing in deaf humans (e.g., Benetti et al., 2017, 2021; Bola et al., 2017) and non-human animals (e.g., Lomber et al., 2010).

Neuroplastic changes have been observed after a congenital, early, and even late onset of sensory deprivation (for deafness, see, e.g., Allman et al., 2009; Sandmann et al., 2012). However, compared to developmental plasticity, the impact of adult plasticity is significantly reduced (for reviews see Bavelier and Neville, 2002; Heimler et al., 2014). Behavioral differences between sensory-deprived individuals and control groups have been associated with changes on the neural level (Gilbert et al., 2001). An impactful example comes from the animal model. Congenitally deaf cats outperformed hearing cats in visual localization of peripherally presented stimuli and visual motion detection. These superior skills could be linked to changes in the posterior auditory field (PAF) in deaf cats, which is associated with auditory localization in hearing cats (Lomber et al., 2010). Changes in auditory areas of deaf cats were also identified at the level of neural layers, which were thinner than in hearing cats (Berger et al., 2017). To understand the underlying modulations, it is crucial to distinguish between different perceptual functions, such as spatial and temporal processing (Cardin et al., 2020). For example, congenitally blind individuals have been shown to outperform sighted controls in tactile temporal order judgment tasks but have displayed deficits in spatial abilities (e.g., Röder et al., 2004). In general, sensory deprivation has been associated with three possible behavioral outcomes: (1) *Hyper-compensation,* that is, better performance, (2) *crossmodal compensation*, resulting in no behavioral differences, and (3) lower performance, supporting the *perceptual deficiency hypothesis* (for a review see Pavani and Röder, 2012).

Furthermore, when examining how individual experiences impact neural organization, considering the role of timing during ontogeny is crucial. The development of perceptual, cognitive, and socio-emotional skills is characterized by specific and limited time windows. For typical progress, certain input must be received within these sensitive and critical periods (Knudsen, 2004). Critical periods have been identified for the development of the sensory systems, such as the auditory system (Sharma et al., 2002; Kral, 2013). Studies in early deaf children who received a cochlear implant (CI) have suggested that for the development of the auditory system, the first 7 years of the lifespan are critical (Weber-Fox and Neville, 1996; Putzar et al., 2007). Other studies have defined an even earlier time window, recommending implantation before the age of 3.5–4.0 years, but not later than 7 years of age (Sharma et al., 2002). Critical periods also exist for higher cognitive functions, such as specific language functions (for a review see Kuhl, 2011). In the case of severely delayed exposure to a first language, some language functions, such as complex syntactic structure, might be irreversibly lost (e.g., Mayberry et al., 2002). Notably, other linguistic features—for example, semantic processing—seem to be less susceptible to critical periods, and the effects of age of acquisition are very different for first and second language acquisition (Curtiss, 1977; Mayberry and Kluender, 2017).

Thus, due to developmental neuroplasticity and critical periods in ontogeny, age of deprivation onset can be considered a crucial variable in studies on deafness and deafblindness (Knudsen, 2004; Kral, 2013). Furthermore, individual language experiences should be examined. Here, a critical determinant might be the age of exposure to a first language. For some participants in studies on somatosensory processing in deaf and deafblind individuals, this will be a signed language. Signed languages are natural, full, and complex languages, which allow a typical development of language areas in the brain (Mayberry and Kluender, 2017). If acquired from birth, signed and spoken languages mostly recruit the same neural network (Neville et al., 1998; Emmorey et al., 2002; MacSweeney et al., 2002; Mayberry et al., 2011; for a review see Campbell et al., 2008). However, in addition to modality-independent language areas, some specific areas are more active or recruited only for signed languages compared to spoken languages (Emmorey et al., 2007, 2014). This might have an impact on, for example, the lateralization of the neural response to non-linguistic stimuli (Bosworth and Dobkins, 1999; Ferjan Ramirez et al., 2014; Bottari et al., 2020). On the other end of the language acquisition continuum, there are deaf and deafblind participants who have never fully acquired a language. Language deprivation due to delayed exposure to a first language changes the organization of language areas in the brain and may have an impact on other, more basic perceptual functions (MacSweeney et al., 2008; Mayberry et al., 2011). However, this perspective has not always been sufficiently taken into account, rendering some of the outcomes of the existing studies unclear.

## Subsections relevant for the subject

### Deaf participants

For deaf individuals, most of the existing work has focused on the visual system (Neville and Lawson, 1987a,b; Finney et al., 2001, 2003; Bosworth and Dobkins, 2002; Fine et al., 2005; Bottari et al., 2014; Almeida et al., 2015; Dewey and Hartley, 2015). Contrary to the visual modality, the development of the somatosensory system and processing of tactile stimuli in deaf individuals have thus far received significantly less attention (Levänen et al., 1998; Levänen and Hamdorf, 2001; Bolognini et al., 2012; Karns et al., 2012; Hauthal et al. 2015; for the animal model, see Meredith and Lomber, 2011). It has been a matter of debate if auditory deprivation results in perceptual deficits or advantages concerning specific stimulus features (for a review see Pavani and Röder, 2012). Importantly, deaf individuals do not display altered processing skills *per se*, but rather for specific tasks (for reviews see Bavelier et al., 2006; Pavani and Bottari, 2012). Activation of auditory areas in deaf individuals has been mostly, but not exclusively, reported for visual motion stimuli (Bavelier et al., 2001; Finney et al., 2001, 2003; Fine et al., 2005; Dewey and Hartley, 2015). Based on the applied methodology, the studies will be separated into behavioral and neuroimaging studies.

#### Behavioral performance

Behaviorally, the results from different studies do not show a clear pattern. Behavioral enhancements as a result of auditory deprivation, that is, a *hyper-compensation* have been observed in deaf adults in, for example, the detection of tactile frequency changes (Levänen and Hamdorf, 2001) or haptic spatial orientation abilities (Van Dijk et al., 2013a). Earlier studies have also found better performance in deaf compared to hearing children in tactile localization tasks (e.g., Chakravarty, 1968). Levänen and Hamdorf (2001) investigated tactile frequency change detection and frequency discrimination abilities in congenitally deaf participants (*n* = 6; age range: 18–23 years) and a hearing control group (*n* = 6; age range: 22–27 years). All deaf participants were reported to be fluent users of FinSL (Finnish Sign Language), but no further information on their language backgrounds (such as the age of acquisition) is provided. The stimuli consisted of vibrations that were presented to the palm and fingers by a vibrating plastic tube. In the frequency change detection task, participants had to detect deviants with a frequency of 180 Hz, as opposed to 250 Hz standards. In the frequency discrimination task, participants had to decide whether the difference between a changing vibration ranging between 160–250 Hz and a 200 Hz reference stimulus was increasing or decreasing. The difference was decreased as a function of individual response accuracy. The results showed an enhanced tactile sensitivity, that is, better detection rates of the unpredictable tactile frequency changes for congenitally deaf individuals compared to hearing controls. In the task on tactile frequency discrimination, Levänen and Hamdorf (2001) did not observe differences between groups—indicating a *crossmodal compensation* in the deaf group. This is in accordance with results from studies on tactile spatial length discrimination (Bolognini et al. 2012).

Contrary to the findings by Levänen and Hamdorf (2001) on tactile frequency detection, Moallem et al. (2010) did not observe differences between deaf and hearing individuals in mean detection thresholds of tactile stimulation (frequency range: 2–300 Hz) to different fingers (thumb, index finger, middle finger). Importantly, while the included deaf participants (*n* = 9; age range: 18–56 years) were congenitally deaf, their language experiences varied highly. Early languages were reported as “ASL, signed exact English (SEE), Pidgin signed English (PSE), spoken English, cued English, and total communication” (Moallem et al., 2010). Reported language usage of the deaf group is divided into “early” and “current”; no specific ages of acquisition were provided. The control group consisted of hearing participants (*n* = 5; age range: 23–58 years) who had acquired spoken language as a first language. Therefore, in addition to the comparably small sample, the two groups displayed very different language acquisition backgrounds.

The results by Moallem et al. (2010) are in line with those from a study by Heimler and Pavani (2014) on simple tactile detection in early and congenitally deaf participants (*n* = 8; mean age = 34.2 years, *SD* = 5.5) and a hearing control group (*n* = 12; mean age = 28.6 years, *SD* = 2.7). The language background of the deaf participants varied. Two had never acquired a signed language, whereas the other six were late learners of Italian Sign Language (LIS) (age of acquisition range: 7–21 years). Tactile stimulators were attached to the fingertips, the forearms, and the neck (the latter location was investigated in the deaf group only). The groups did not differ in response time, and there was no difference in behavioral performance for the deaf group as a function of tactile stimulation location. Yet another different outcome, that is, lower performance in tactile detection in congenitally deaf (age range: 14–20 years; language backgrounds not reported) compared to hearing participants was reported by Frenzel et al. (2012).

Another example of better tactile performance in deaf compared to hearing individuals was reported by Van Dijk et al. (2013a). In this study, a haptic spatial orientation task was presented to congenitally deaf signers (*n* = 15; mean age = 41.4 years, age range: 19–66 years), hearing sign language interpreters (*n* = 16; mean age = 38.4 years, age range: 26–51 years), and hearing controls (*n* = 16; mean age = 44.8 years, age range: 26–57 years). All deaf participants had acquired a signed language (Sign Language of the Netherlands, NGT) as their first language; all hearing signers had a bachelor’s degree in interpreting, three had deaf parents and grew up with NGT from birth. The signing skills of the other hearing interpreters were described as “near native” (no additional language assessment tasks were performed). The participants were blindfolded and asked to set a test bar parallel to a reference bar with an orientation of 0°, 30°, 60°, 90°, 120°, or 150°. Both bars were placed on a table in front of the participants. They first touched the reference bar with their right hand and, after a delay of 2 or 10 s, respectively, adjusted the test bar accordingly with their left hand. The results revealed better haptic spatial orientation processing skills in the deaf group than in hearing signers and non-signers.

Notably, in a second study from the same authors including a tactile spatial configuration task, sensory experience was not the critical determinant for altered behavioral performance (Van Dijk et al., 2013b). Here, enhanced somatosensory processing was observed as a result of the acquisition and usage of NGT instead. Based on the participants’ background information, it can be assumed that the task was presented to almost the same sample as in Van Dijk et al. (2013a): early deaf individuals (*n* = 15; mean age = 41.4 years, age range: 16–66 years), hearing interpreters (*n* = 16; mean age = 38.4 years, age range: 26–51 years), and hearing controls (*n* = 16; mean age = 44.8 years, age range: 26–57 years). The experiment consisted of three parts, divided into five trials. In the first part (trials 1–3), the blindfolded participants had to match 10 haptically presented shapes to cut-outs on a wooden board, which they had not seen before. In the second part (trial 4), the shapes had to be placed in their previous positions on a board without cut-outs. In the third part (trial 5), the wooden board with cut-outs was rotated while the shapes had to be placed again. Reaction time was measured for the first and the third part of the study. Results revealed that the deaf and hearing signers were significantly faster and outperformed the hearing non-signers in trials 1–3 and 5. This indicates that it is not only the sensory deprivation but also language experience which can shape the processing of touch in individuals. No differences between groups were observed in the second part of the study, (trial 4).

Some studies on somatosensory processing in deaf individuals have supported the *perceptual deficiency theory*, which states that the loss of one sensory modality will negatively impact the spared modalities (for a review see Pavani and Röder, 2012). This perspective has been supported by, for example, studies on temporal discrimination of tactile stimuli, in which deaf individuals performed significantly worse than hearing controls (e.g., Heming and Brown, 2005; Bolognini et al., 2012; Papagno et al., 2016). In a study on temporal detection skills, Heming and Brown (2005) presented tactile and visual stimulation to congenitally and early deaf individuals (*n* = 20; mean age = 22.44 years, age range: 18–31 years) and a matched hearing control group (*n* = 20; mean age = 22.70 years, age range: 18–32 years). All deaf participants reported American Sign Language (ASL) as their first language—however, no further information on language acquisition and usage was provided, and it is possible that some did experience delayed language acquisition. In the tactile task, participants had to detect if two mechanical tactile stimulations presented to the index and the middle finger of the left, right or both hands occurred simultaneously or not (in fact, the stimuli were never presented fully simultaneously). The results revealed significantly higher temporal detection thresholds for the deaf group than the hearing group (deaf group: mean = 84.18 ms, *StD* = 25.34 ms; hearing group: mean = 21.59 ms, *StD* = 14.99 ms).

Bolognini et al. (2012) also addressed the question of how tactile abilities will be impacted by auditory deprivation. To this end, they presented tactile stimuli in two different tasks (temporal and spatial) to groups of congenitally deaf individuals and hearing controls. In the temporal task, nine deaf participants (mean age = 41 years, age range: 25–52 years) and nine hearing controls (mean age = 38 years, age range: 27–60 years) were included. Seven of the deaf participants had acquired LIS before the age of 3 years because they had one or two deaf parents or attended an institution in which LIS was used. The other two deaf participants had not acquired a signed language. No further details on individual language backgrounds were provided. For the spatial task, seven deaf and seven hearing individuals participated. The mean age was 44 years for the deaf group (age range: 25–53 years) and 32 years for the hearing controls (age range: 24–49 years). Five of the deaf participants were early signers (<3 years), and the other two were non-signers. Vibrotactile stimuli were attached to the index fingers of both hands. In the temporal task, participants discriminated stimuli with a duration of 15 ms or 25 ms, respectively (with interrupting pulses after each 5 ms). The stimulation was presented to the fingertips of the index fingers. In the spatial task, the participants discriminated the spatial length of the stimulation, which was presented to either two or three points on the index fingers. Behavioral results on perceptual sensitivity revealed that the hearing controls significantly outperformed the deaf group in the temporal task, whereas no differences between groups were observed in the spatial task.

In a different kind of temporal task, Sharp et al. (2018) found an altered performance in congenitally deaf individuals (*n* = 13; mean age = 38.4 years, age range: 29–57 years) compared to hearing controls (*n* = 13; mean age = 33.4 years, age range: 20–59 years) in a temporal order judgement task (TOJ). Tactile stimulation was delivered through a small foam cube that was held between the thumb and index fingers of both hands. The stimulus onset asynchrony (SOA) varied and was ±400, ±200, ±100, or ±50 ms. Participants had to decide on which side the stimulation was presented first (left or right; negative SOA values for trials in which the stimulation was presented on the left hand first). The experiment included conditions with uncrossed and crossed arms. Compared to the hearing controls, the deaf group showed significantly higher error rates in the blocks with crossed arms. The authors concluded that the deaf individuals were less successful in managing the “conflict between visual and somatosensory body-related information through a change in posture” (Sharp et al., 2018). Notably, though, 12 of the deaf 13 deaf participants used spoken language, whereas only one of the participants primarily communicated in a signed language. No information is given about the deaf participants’ language acquisition history and, thus, it is not possible to disentangle the impact of deafness vs. language experience.

In a study including a visuo-tactile TOJ task with a crossed-arms condition, Scurry et al. (2020a) tested early deaf (*n* = 12; mean age: 41.73 years) and matched hearing participants while they recorded the EEG. Here, the results did not reveal accuracy differences, that is, temporal order discrimination and perceived synchrony of the visuo-tactile stimulation. Importantly, while the authors provide detailed information about the etiologies of the participants, this is not the case for their language backgrounds. The observation of crossmodal compensation is in line with other studies that have reported similar behavioral outcomes for deaf and hearing groups in temporal processing (Bross and Sauerwein, 1980; Poizner and Tallal, 1987; Nava et al., 2008; Moallem et al., 2010). For example, Moallem et al. (2010) did not find group differences in tactile temporal processing skills of congenitally deaf individuals and hearing controls. In their task, stimuli of 50 Hz at the thumb and 250 Hz were delivered at the index finger and either asked which stimulus was preceding the other one or which was presented later, respectively. There was a high amount of individual variability in behavioral outcomes in both groups, especially the deaf group.

While somatosensory processing has not yet been extensively investigated in deaf individuals, even less is known about the interaction of the spared senses as a consequence of auditory deprivation. In the first study on visuo-tactile processing in congenitally deaf individuals (*n* = 13; all participants reported having a family history of congenital deafness and acquired ASL in childhood), Karns et al. (2012) observed evidence for altered multisensory processing compared to hearing controls as well as reorganization of auditory brain regions. In an fMRI study, they presented a touch–induced flash illusion—a single flash is perceived as two flashes if two tactile stimuli are presented at the same time, analogously to the sound-induced double flash illusion (Shams et al., 2000; Violentyev et al., 2005). Only the deaf individuals were susceptible to the illusion for reduced auditory-tactile interactions in congenitally blind individuals compared to sighted controls, see (Hötting et al., 2004; Hötting and Röder, 2004). This was interpreted as a stronger multisensory interplay between vision and touch as a result of deafness (Karns et al., 2012).

Hauthal et al. (2015) presented a speeded detection task with visual, tactile, and crossmodal stimulation while they recorded the EEG. The findings revealed a reduced redundancy gain in response times (RTs) for crossmodal versus unimodal stimulation in congenitally and early deaf individuals (*n* = 10; mean age = 43, *SD* = 7 years, age range: 36–57 years) compared to hearing controls (*n* = 10; mean age = 43, *SD* = 9 years). The deaf participants were all signers of German Sign Language (DGS), however, the age of acquisition varied and only one participant had acquired DGS from birth.

In a recent EEG study investigating visuo-tactile motion processing, Villwock et al. (2022) observed a higher false alarm rate for incongruent motion stimuli in congenitally deaf native signers of DGS (*n* = 21; mean age = 26.14 years, age range: 19–48 years) compared to matched hearing non-signers. The tactile motion was presented to the index fingers of both hands; the visual motion was presented *via* adjacently located LED lights. Participants were asked to detect deviants with an interrupted movement which were going in a target direction. Importantly, for the deviant stimuli, only one of the modalities (visual or tactile) was interrupted. The false alarm rate did not differ between groups in the congruent condition, indicating different stimulus selection strategies in congenitally deaf signers compared to hearing non-signers.

#### Neuroscientific results

Only a few neuroscientific studies have investigated somatosensory processing in deaf individuals, and some reported responsiveness of auditory areas to tactile stimulation that was not present in hearing controls (Levänen et al., 1998; Auer et al., 2007). Evidence of crossmodal plasticity—that is, enhanced activation of auditory areas following tactile stimulation in deaf individuals compared to hearing controls—has been found in studies applying magnetoencephalography (MEG) (e.g., Levänen et al., 1998) as well as functional magnetic resonance imaging (fMRI) (e.g., Auer et al., 2007; Karns et al., 2012). Furthermore, single-unit recording studies have reported responsiveness of neurons in auditory cortices of deaf non-human animals following both somatosensory and visual stimulation (e.g., Allman et al., 2009; Meredith and Allman, 2009, 2012; Meredith and Lomber, 2011; Land et al., 2016).

In a single-case magnetoencephalogram (MEG) study including tactile stimulation to the fingers and the palm of the left hand, Levänen et al. (1998) observed responses in primary auditory areas of a senior deaf participant (age = 77 years), but not in a group of hearing control participants (*n* = 6; age range: 26–37 years). Moreover, the MEG analysis revealed specific responses in auditory areas to 180 Hz vs. 250 Hz, respectively. The congenitally deaf participant was a signer of Finnish Sign Language (FinSL) and came from a family with five deaf siblings. Thus, the results might indicate changes due to neuroplasticity after auditory deprivation—and/or as a result of FinSL acquisition.

Indication of crossmodal reorganization was also found in an fMRI study by Auer et al. (2007). The fMRI has a better spatial resolution than MEG. Here, the sample was more balanced than in Levänen et al. (1998) and consisted of six deaf participants (mean age = 23 years, age range: 19–26 years) and six hearing controls (mean age = 24 years, age range: 19–31 years). Two of the deaf participants were congenitally deaf; the etiology of the other participants was unknown. All reported to have used hearing aids in the past; information on language backgrounds is not provided. The vibro-tactile stimulation was either derived from speech or at a fixed frequency of 125 Hz sine waves and presented at the right thumb. The activation of auditory areas was significantly stronger and more widely distributed in the deaf individuals than in hearing controls for both types of stimulation (Auer et al., 2007).

In their study on temporal and spatial tactile perception in congenitally deaf individuals and a hearing control group, Bolognini et al. (2012) examined the effect of deafness on the processing of touch. They applied transcranial magnetic stimulation (TMS) to monitor the timing of involvement of the primary somatosensory area (S1) and Superior Temporal Gyrus (STG) as a function of the somatosensory processing. Behaviorally, the hearing group outperformed the deaf group in the temporal task, but no such group differences were observed for the spatial task. The TMS results showed that in addition to primary somatosensory areas (S1), both tasks involved activation in the auditory association cortex at a time window of 60–120 ms in deaf individuals. For hearing controls, a similar pattern was observed in SI, however, the STG displayed involvement at a later latency of 180 ms, and only for the temporal task. For the hearing group, this area was involved at a later latency (180 ms) and for the task of temporal discrimination only (for hearing participants see also Bolognini et al., 2010). Moreover, analyses revealed a correlation between temporal discrimination task performance and the effect of disruption by STG-TMS at 180 ms: The better the behavioral performance, the larger the disruption induced by the STG-TMS at that time point. In a recent fMRI study, Zimmermann et al. (2021) also found different results for temporal vs. spatial somatosensory processing in a group of deaf and hard-of-hearing participants (*n* = 21). All participants were signers of Polish Sign Language (PJM), and their age of acquisition ranged from birth to primary school entry. While both stimulus types resulted in the recruitment of auditory cortex, this was task-specific for the temporal task only. On the contrary, spatial stimulation evoked activation in auditory areas regardless of the experimental task. Moreover, the activation was more widely spread for spatial compared to temporal processing.

Contrary to these results, Hickok et al. (1997) did not find indicators of crossmodal plasticity in an MEG study with one congenitally deaf participant (28 years old), who had acquired ASL from birth. Although the observed activity patterns were compared with those of hearing control participants, a control group is not specified. The stimulation included visual and tactile stimulators, and a motor task (self-paced finger-tapping) was conducted. No behavioral task was included. Tactile stimulation consisted of mechanical taps (17–20 psi, duration = approx. 30 ms) that were presented at digit segments, the lip, and the tongue. The results did not reveal activation in auditory areas, responses were observed in visual, somatosensory, and motor areas.

While to date, somatosensory processing has not been thoroughly investigated in deaf individuals, even less is known about the neural patterns regarding the interplay of vision and touch in deaf individuals (Karns et al., 2012; Villwock et al., 2022). In the first human study on multisensory processing in congenitally deaf individuals, Karns et al. (2012) used fMRI to examine visual, tactile, and visuo-tactile stimulation processing. The stimuli were static, tactile stimulation consisting of air puffs presented to the face. The fMRI results showed evidence for enhanced multisensory processing associated with a reorganization of auditory brain regions. Deaf individuals showed significantly stronger activation of primary auditory cortices (Heschl’s gyrus) than hearing controls for all stimulus types. Responses in Heschl’s gyrus were more enhanced for crossmodal and tactile stimulation than for visual stimulations. The response in superior temporal sulcus (STS) was comparable for the visual modality. Moreover, it was only the deaf group that was susceptible to a touch-induced double-flash illusion, and the strength of the illusion was positively correlated to the associated signal changes in auditory cortices. The results point to altered multisensory processing and crossmodal reorganization after congenital deafness (Karns et al., 2012).

In an fMRI study on tactile motion processing, Scurry et al. (2020b) tested seven early deaf participants (age range: 31–55 years) and matched hearing controls (age range: 28–54 years). Language backgrounds were not reported. Tactile stimuli were presented to the right index finger and included four different directions of motion: Up, down, left, and right (each presented for 2 s). Participants performed a behavioral task to ensure their attention to the stimuli, however, behavioral results were not reported. The results of a population receptive field analysis revealed a comparable neural response of the groups to tactile motion in primary and secondary somatosensory cortices. However, compared to hearing controls, the deaf group displayed a lower proportion of directionally tuned voxels in primary somatosensory cortex. Furthermore, they showed larger responses to tactile motion in the right posterior superior temporal sulcus (pSTS), pointing to crossmodal plasticity as a result of the early auditory deprivation. This is in accordance with findings from González-Garrido et al. (2017), who presented an oddball task with vibrotactile stimuli to early deaf participants (*n* = 14; mean age = 21.96 years, SD = 6.63 years) and matched hearing controls (*n* = 14; mean age = 21.93 years, SD = 5.02 years). Except for one native signer, all deaf participants acquired Mexican Sign Language (LSM) after the age of 7 years. To investigate how the somatosensory system might be a substitute for auditory input and support the perception of speech, training with sound wave stimuli (five 1-h long sessions) was conducted. The training stimuli were targeting pure tone frequency and duration discrimination, but also complex natural sounds. Stimulation was presented to the right index finger. The oddball task with 700 and 900 Hz pure tones (80% standards, 20% deviants) was performed pre-and post-training. ERP analyses revealed differences in the topography of the electrophysiological response between groups. In a time window comprising the P3 wave, a right lateralized response was observed for the deaf, but not the hearing group.

In an EEG study investigating visual, tactile, and crossmodal static stimulation in a simple detection redundant target task, Hauthal et al. (2015) observed a shorter latency of the N200 for visuo-tactile in comparison to unimodal tactile conditions in hearing participants compared to a group of congenitally deaf native signers. This might suggest an altered and delayed multisensory processing as a consequence of congenital deafness (Hauthal et al., 2015). However, the deaf group also displayed significantly shorter N200 latencies than the hearing group in the unimodal tactile condition—presumably leading to a larger difference between visuo-tactile and tactile latencies in the hearing group. Hauthal et al. (2015) observed a delayed latency of the N200 in deaf individuals compared to hearing controls for visuo-tactile in comparison to unimodal tactile conditions, possibly suggesting altered multisensory processing as a result of auditory deprivation.

In another recent EEG study, Scurry et al. (2020a) examined temporal processing skills in early deaf participants and hearing controls. They employed a TOJ task with visual and tactile stimuli. There were no behavioral differences between groups. However, the ERP results displayed larger amplitudes of both the visual P100 (for all SOA levels) and the tactile N140 (for the shortest asynchronous presentation at ±30 ms as well as synchronous stimuli) in deaf compared to hearing participants. Furthermore, the deaf group showed a longer latency in the somatosensory P200 than the hearing control group.

Villwock et al. (2022) presented congruent and incongruent visuo-tactile motion stimuli to congenitally deaf first language and first modality (L1M1) signers, and hearing controls. The ERP results showed a delayed congruency of motion effect in the deaf group compared to hearing controls (200–280 ms vs. 348–448 ms after stimulus onset, respectively), and thus, do not point to enhanced motion direction-specific interactions between the visual and tactile system. The lateralization of the congruency effect was opposed for the groups—the deaf group showed a left lateralized response, whereas the effect was right lateralized in the controls. Moreover, ERPs between 140 and 164 ms were more anteriorly distributed in the deaf than in the hearing group, possibly indicating activation in auditory areas as a consequence of crossmodal plasticity.

#### Summary: deaf participants

To conclude, there are comparably few studies on the impact of deafness on the somatosensory system, and both the processing of touch and the crossmodal interplay of the spared modalities are not well understood. So far, studies have revealed different results in performance and neural responses to stimulation, including the lateralization of activation. These inconsistencies might be partly based on the specific tasks in the studies. Furthermore, they might be due to highly heterogeneous samples of deaf participants both between and within studies. So far, deaf participants with very different backgrounds are often assigned to the same group. This includes congenitally vs. early and late deaf individuals, and highly different backgrounds regarding language acquisition. In some cases, in which the deaf samples were rather homogeneous regarding their sensory experiences, language experience was not taken into account (e.g., Heming and Brown, 2005). The studies which considered both factors (e.g., Villwock et al., 2022), have mostly focused on congenitally deaf L1M1 signers and compared them to non-signing hearing control groups. As a result, the impact of sensory deprivation vs. the usage of a signed language cannot be fully distinguished, impeding an unambiguous interpretation of the results.

### Deafblind participants

Including the group of deafblind individuals allows crucial insights into the consequences of audio-visual deprivation on the perception of touch. Importantly, individual etiologies for deafblindness are highly heterogeneous (Dammeyer, 2013), a factor that must be taken into account when considering the number and outcome of existing studies. To date, there is little insight into how somatosensory processing in deafblind individuals might be altered as a function of the specific sensory deprivation and individual language experience. Compared to unisensory information, a combination of different sensory modalities usually results in enhanced performance, e.g., for response times (Meredith and Stein, 1986; Stein et al., 2010). This may be due to the supramodal characteristics of an event—e.g., space and time—which can be simultaneously coded by the different sensory systems. Unimodal stimulation is seen to be relatively weak compared to a multimodal percept (Stein et al., 2010), and multisensory neurons have been found in different brain regions in individuals (for reviews see Stein and Stanford, 2008; Murray et al., 2016). However, so far, it remains inconclusive how the brain organizes itself as a function of a bisensory deprivation such as deafblindness. Furthermore, it remains unknown if and how the sensory processing of congenitally deafblind individuals might differ from congenitally deaf individuals, who became blind later in life.

The sensory experiences of deafblind participants differ due to their etiology. There are numerous possible reasons for deafblindness in humans, resulting in high variability of individual experience. Etiologies can include pre-, peri-and postnatal causes. This includes, *inter alia*, congenitally deafblind individuals and those who were born deaf, but experienced a later onset of blindness (e.g., due to *Usher syndrome;* Vernon, 1969). The exact number of deafblind individuals in a community is often unknown. For example, based on information from community members, the German deafblind community might consist of approximately 10,000 members, most of them seniors. A previous study in Denmark provided an estimate for a prevalence of 1: 29,000 for congenital deafblindness in the Danish population. Late deafblindness has a significantly higher frequency in seniors compared to children and younger adults. In general, there is a larger number of older deafblind people than deafblind children and young adults (Dammeyer, 2010, 2013).

While linguistic experiences in the Deaf Community display a considerable degree of variance, the situation is even more complex for deafblind individuals, and individual language backgrounds are highly diverse (Mesch, 2001; Willoughby et al., 2014; Edwards and Brentari, 2021). This is partly due to the heterogenous nature of deafblindness, such as the age of onset of blindness. Exposure to language in this sample can also depend on coexisting intellectual conditions. Furthermore, expressive and receptive communication channels might differ, for example, based on individual motor skills. Language acquisition and usage can include, *inter alia*, early or delayed acquisition of a signed language, a signed communication system, a tactile sign language, or tactile systems such as *Lormen* (also known as the *Lorm-Alphabet*), in which single letters are written onto the hand of the communication partner. Some deafblind individuals use Braille as a tactile writing and reading system. Some communicate in spoken language or may never have fully acquired a language. Regarding speech, the Tadoma method can be used to convey language input. To this end, the deafblind person touches the face of the speaker (Reed, 1996). However, this method is rarely used nowadays. For communicating in a tactile sign language, the receiver touches the hand(s) of the person who is producing the signs (Mesch, 2001). In some communities and their tactile sign languages, there is a stronger preference to follow the dominant hand only, and the positions of the hands differ between languages (Mesch, 2011, 2013; Willoughby et al., 2018). More recent research has begun to examine the emergence of a new tactile language system in the United States, called *Protactile*. Deafblind signers adapt ASL when using it through the tactile channel, resulting in the emergence of new grammatical systems in the tactile language (Edwards, 2018; Edwards and Brentari, 2020, 2021).

One might assume that the acquisition and usage of a tactile language will increase non-linguistic processing. However, to date, the specific consequences of tactile language use on other tactile perceptual processing remain unknown.

#### Behavioral performance

Most studies have investigated deafblindness on the single-case level (e.g., Kawasaki et al., 1997; Janssen et al., 2007; Obretenova et al., 2010). For example, Kawasaki et al. (1997) investigated speech processing in a 74-year-old deafblind woman, who had just received a cochlear implant (CI) in her left ear. At the time of the implantation, the participant had been blind (due to retinal detachment in both eyes) and deaf in her right ear (due to a sudden hearing loss) for 9 years. Moreover, 2 years before implantation, she experienced a profound hearing loss in her left ear as well. The CI-implantation was conducted unilaterally in the left ear. Results at two and 18 months after implantation showed high vowel and consonant discrimination rates. Previous studies on cochlear implantation in deafblind individuals reported similar results (Martin et al., 1988; Ramsden et al., 1993), and the authors conclude that deafblind participants might have an outcome of the CI “similar to or better than the many sighted cochlear implant patients” (Kawasaki et al., 1997). However, it is important to note that none of these single-case studies on CI-implantation included congenitally deaf participants.

Janssen et al. (2007) focused on perceptual instead of speech processing in their single-case study. They presented a tactile perception (shape discrimination) and a memory task to a congenitally deafblind woman (40 years old) and eight hearing and sighted controls (mean age = 34.75; age range: 20–60 years). Participants in the control group were blindfolded and got noise-shielding headphones. There was no detailed report of the deafblind participant’s language experience, however, the authors state that regarding informed consent, the participant, her parents, and her caregiver gave “oral and written permission” (Janssen et al., 2007). The experimental task was explained by an interpreter “through means of finger spelling in her hand” as well as “‘natural gestures’ (such as pointing or other commonly used hand gestures)” (Janssen et al., 2007). The results revealed an average response time of 5.3 s for the perception task and 3.2 s for the tactile memory task for the deafblind participant. Numerically, this was faster than any participant in the control group. However, when encoding speed in the memory task was taken into account, no processing advantage in the deafblind participant was observed. Moreover, more errors than in the control group occurred. No further statistical tests were performed.

Arnold and Heiron (2002) examined tactile memory in deafblind (*n* = 10; mean age = 58.8 years, age range: 35–92 years) and a sighted and hearing control group (*n* = 10; mean age = 51.4 years, age range: 25–64 years). Participants were asked to rate their current degree of deafness and blindness on a scale from 1 to 5. The ratings showed a rather high variation in sensory experiences on the individual level. Mean ratings for the degree of the sensory deprivation were 4.1 for blindness (*SD* = 1.10; range: 2–5) and 3.5 for deafness (*SD* = 0.85; range: 3–5). Only one of the deafblind participants reported an onset of deafness and blindness from birth. The etiologies of the other nine deafblind participants were not described in detail, though one of them is reported to have been diagnosed with Usher Type 2. Four tasks were conducted: A recognition task including 12 toy animal shapes, a recognition task including domino tiles, a spatial recall task, and a spatial task including matching pairs of textures on cards. Hearing controls needed more time than deafblind individuals to remember the items. Contrary to expected better performance in the deafblind participants, behavioral analyses did not reveal group differences in any of the tasks regarding accuracy. The authors suggest that this might be because, with one exception, the deafblind group included late deafblind individuals. The deafblind participants were reported to be either retired or “registered disabled” and participants’ individual language experiences might have varied highly. There was no mention of language use in this group, however, the control group consisted of “five volunteers who worked with the deaf-blind on a part time basis, and five who worked in industry”. The ones working with deafblind individuals had varying levels of knowledge of “the deaf-blind manual sign language” (Arnold and Heiron, 2002). Notably, the age range in the deafblind group was larger than in the control group, and language experience was not a factor that was taken into account.

A different outcome was reported in another study with a comparably high number of participants. Papagno et al. (2016) presented a spatial and a temporal tactile task to deaf (*n* = 7), blind (*n* = 7), deafblind (*n* = 7), and hearing and sighted control participants (*n* = 14). For one deafblind participant, the etiology was not clear. Due to Usher Syndrome (type 1), the other six deafblind participants had all become blind in early adulthood (mean age of onset of blindness = 16.28; range: 1–40 years); no congenitally deafblind individuals were included. One participant primarily communicated *via* the tactile Malossi system, in which letters are written on the hand. The other six deafblind participants used tactile LIS (LISt). As in Arnold and Heiron (2002), the deafblind group was older than the other groups (mean age: deafblind = 62 years, age range: 40–74 years; deaf = 45 years, age range: 27–51 years; blind = 39 years, age range: 24–50 years; controls = 44 years, age range: 28–67 years). Importantly, although being congenitally deaf, none of the deaf participants reported having acquired a signed language from birth. Only two out of seven deaf participants had learned a signed language at all—in this case, LIS. No information is provided about the specific kind of linguistic experience the other five deaf individuals had. However, all participants were reported to be using hearing aids and to be “almost fluent Italian speakers” (Papagno et al., 2016).

For the temporal task, tactile standard and target stimuli were presented to the tips of either the left or the right index finger. Target duration was 25 ms. This included three pulses of 5 ms, separated by two inter-pulse intervals (IPIs) of 5 ms. Standard duration was 15 ms, interrupted by two 5 ms long IPIs. Participants were asked to respond verbally or manually indicate if they perceived a target stimulus. In the spatial task, stimulation was presented at the index fingers and through two vibrotactile stimulators for standards and three for target stimuli. Stimulus duration was 5 ms. The deaf and deafblind groups showed better results in the spatial than the temporal task, whereas both the blind group and the control participants were better in the temporal task. Deaf and deafblind individuals displayed lower performance for temporal discrimination than the controls. The deafblind group performed better than the blind group in the spatial task. Overall, the authors concluded that the results indicate that sensory deprivation does not result in better tactile performance (Papagno et al., 2016). Again, due to the sample, it cannot be clarified whether these findings are based on sensory deprivation, linguistic experience, or both.

In a study with more complex tactile stimuli, Papagno et al. (2017) presented a short-term memory task to deaf (*n* = 16, mean age = 49.34 years, median age = 49.5, age range: 26–78 years), blind (*n* = 15; mean age = 49.34 years, median age = 56, age range: 24–77 years), deafblind (*n* = 13, mean age = 56.15 years, median age = 66, age range: 21–75 years) and sighted and hearing participants (*n* = 13; median age = 67 years). There was no difference between groups regarding age (*p* = 0.54) and years of education (*p* = 0.54). Degrees of deafness and blindness varied (severe deafness: 71–95 dB, profound deafness: >96 dB1 dB; blindness: partial, that is, with a residual visual acuity of 1/20; and total, with no light reception), and so did individual etiologies. Etiologies of deafblind participants included, *inter alia*, Usher syndrome, Poliomyelitis, Norrie syndrome, KID syndrome, and repeated otitis. No congenitally deafblind individuals were included. All but one deafblind participant were users of Braille (mean age of acquisition = 9.25 years; range: 1–24 years). None of the deaf and deafblind participants had acquired a (tactile) signed language from birth; some participants never acquired one. The mean age of acquisition for LISt in the deafblind group (LISt users: *n* = 8) was 12.59 years (range: 6–35 years). Mean age of LIS acquisition in the deaf LIS users (*n* = 14) was 6.5 years (range: 3–20 years).

To examine short-term memory, the authors presented a task with checkerboard patterns of different sizes with either rough or smooth surfaces. Participants were presented with three patterns for each size (starting with the smallest) for 10 s and then asked to recreate the pattern. The experimental session ended if a participant did not pass two out of the three trials. Behavioral measures included completion time, number of correctly filled matrices, size of the largest completed matrix, and tactile span. The results revealed no difference between blind, deafblind, and deaf participants. In contrast to other studies (e.g., Arnold and Heiron, 2002), there was no difference between those groups and the control group regarding completion time. The deaf and the blind group outperformed the controls in all other behavioral measurements, whereas the deafblind group only showed a statistical tendency for better performance in the number of correctly reproduced matrices (*p* = 0.063). Performance and age of acquisition of Braille were negatively correlated in the deafblind and blind groups, pointing to an impact of Braille experience on tactile short-term memory skills. Notably, LISt acquisition, the onset of deafness (in deaf and deafblind participants), and blindness (in blind and deafblind participants) were not correlated with task performance. No correlation analysis including LIS acquisition was reported.

Most deafblind participants from Papagno et al. (2016, 2017) also participated in a behavioral study by Cattaneo et al. (2018), investigating bilateral haptic spatial attention. While a group of early deaf individuals (signers of LIS and non-signers) did not show a bias to shift to the left or right side from a veridical midpoint in the line bisection task, deafblind participants displayed a bias to the left side. This result was in accordance with the behavioral outcomes of early blind participants as well as a hearing and sighted control group. This points to different processing mechanisms as a function of unisensory and bisensory sensory deprivation, respectively, and the impact of visual experience in the deaf individuals.

#### Neuroscientific results

Osaki et al. (2004) examined the processing of tactile words and non-words in an MEG study with a 38-year-old male participant, who had become deafblind at the age of 35 years. The participant had started learning a tactile language (presentation of Japanese characters to the hand) two years before the study took place. His data was compared to six hearing and sighted controls (mean age = 30.3 years, age range: 24–45 years). During the session, nouns (comprising three characters) and non-words were presented to the right hand. The analyses revealed activation in left IFG, left middle occipital gyrus, and left posterior superior temporal gyrus following the tactile word condition—but not the non-word stimuli—in the deafblind participant. These results were confirmed by an additional positron emission tomography (PET). The hearing participants showed varying patterns of activation in the same areas after being presented with tactile words.

In a single-case fMRI study, Obretenova et al. (2010) examined the neural processing of Braille, Print on Palm (POP), and haptic ASL (hASL). The deafblind male participant was born deaf and became blind at the age of 6 years (due to bilateral ocular trauma). For communication, he used Braille, POP, and hASL which he started acquiring around the age of 10 years. A 24-year-old, hearing and sighted female was recruited as a control participant. She reported having had 3 years of experience with hASL due to training to become an ASL interpreter for deafblind individuals, but she did not know POP or Braille. For the deafblind participant, each of the three input types (Braille, POP, hASL), words, and non-words were presented to the left hand (as preferred by the participant). Moreover, the experimental conditions included rest as well. For the control participant, only hASL (and rest) were investigated. Each trial was 3 s long (with six presentations per block). As an experimental task, participants were asked to decide whether the presented words started with a consonant or a vowel. There were no differences between the participants regarding behavioral performance in hASL (both achieved an accuracy of 78.1%). For the deafblind participant, the fMRI results showed enhanced activation for the three input types in left inferior frontal and posterior superior temporal language areas. Moreover, the deafblind participant displayed increased bilateral activation in occipital cortex. A diffusion tensor imaging-based tractography revealed stronger connectivity between occipital and temporal areas in the deafblind participant. The control participant showed increased activation following hASL input in left inferior frontal areas and, although less strongly, in posterior superior temporal language areas. However, no comparable increase in activation was observed in occipital areas. These findings are consistent with the assumption of crossmodal plasticity as a result of sensory deprivation in the deafblind participant (see Auer et al., 2007; Bedny et al., 2011).

#### Summary: deafblind participants

For deafblind individuals, the lack of research on somatosensory processing is even more substantial than for deaf individuals. Overall, the consequences of deafblindness on the processing of touch remain mostly inconclusive, and sometimes, findings from different studies are providing conflicting information. Moreover, for this group, it is particularly important to consider the impact of possible comorbidities. One might expect a difference as a function of sensory and/or linguistic experience, but at this point, this assumption remains partly speculative. To identify the impact of individual experience on brain development and neuroplasticity, different groups of individuals should be identified and tested in similar experimental paradigms. Notably, while the studies with deaf individuals focused on congenitally and early deaf participants, the literature review on deafblind individuals was mostly limited to research concerning individuals who experienced a late onset of (deaf)blindness.

## Discussion

This review addresses existing studies on the processing of touch and associated crossmodal plasticity in deaf and deafblind individuals. To date, little is known about the development of the somatosensory system in these groups. Regarding the consequences of deafness, the processing of touch has received less attention than the visual system. Even fewer studies exist that are investigating the sensory development of deafblind individuals, for whom touch is the only sense that can ensure communication. Some of the few published studies to date point to an altered processing as well as a crossmodal reorganization in both deaf and deafblind individuals (e.g., Levänen et al., 1998; Obretenova et al., 2010; Karns et al., 2012). These changes are expressed on behavioral and neural levels. Thus, deafness and deafblindness appear to impact somatosensory (and in the case of deafness, also multisensory) processing to some extent. However, studies on visual processing in deaf individuals have shown that sensory deprivation does not result in general enhancements or deficits of processing abilities in the remaining senses. Instead, group differences depend on stimulus features and the investigated perceptual functions. For example, deaf participants have been shown to outperform hearing groups in simple detection, but not discrimination of visual stimuli (for reviews see Bavelier et al., 2006; Pavani and Bottari, 2012). Moreover, considering motion as a relevant stimulus feature for early deaf individuals is supported by previously observed altered visual motion detection abilities compared to hearing controls (Parasnis and Samar, 1985; Neville and Lawson, 1987a; Armstrong et al., 2002; Proksch and Bavelier, 2002). Deaf participants have displayed enhanced behavioral performance and larger neural responses than hearing individuals to peripheral than focal stimulation (e.g., Neville et al., 1983; Neville and Lawson, 1987a; Bottari et al., 2010, 2011). Furthermore, the interplay of the visual and the tactile modality seems to be altered in congenitally deaf signers (Karns et al., 2012; Hauthal et al., 2015; Villwock et al., 2022). Regarding studies including tactile stimulation, different stimuli features and perceptual functions such as spatial and temporal processing have been investigated. However, the findings have not always been consistent, and often, the sample sizes are quite small. This poses a challenge concerning the interpretation of these studies’ results.

Investigating the specific sensory experience of an individual allows the identification of determinants for neuroplastic changes as a function of sensory deprivation. For example, due to the higher degree of neuroplasticity in ontogeny, and the impact of critical periods on the developmental trajectory, the age of deprivation onset is considered a very important factor for perceptual and linguistic development (Knudsen, 2004; Mayberry and Kluender, 2017). In some of the existing studies on somatosensory processing, deaf and deafblind participants were older than controls (e.g., Arnold and Heiron, 2002; Papagno et al., 2016). This must be considered when interpreting results from these studies—previous work has shown higher thresholds in hearing seniors (> 60 years of age), indicating an age effect on these kinds of tactile processing skills (see, e.g., Brown and Sainsbury, 2000).

Importantly, some changes in behavioral outcomes might be associated with language experience instead of the sensory deprivation (see Emmorey et al., 1993, for better performance in visual mental rotation in deaf and hearing signers compared to hearing non-signers). Although studies have shown that the language network is mostly identical for spoken and signed languages, there are some modality-specific differences. For example, complex syntactic processing seems to be modality-independent and can be localized in the anterior and posterior superior temporal sulci (aSTS, pSTS) for both signed and spoken languages (Matchin et al., 2022). On the contrary, the supramarginal gyrus (SMG) is more active for signed than for spoken word production (Emmorey et al., 2002, 2007).

Van Dijk et al. (2013a) observed better tactile spatial configuration abilities for both deaf and hearing signers compared to hearing non-signers. Thus, the critical factor for altered processing here is not sensory deprivation, but language experience (all hearing participants were proficient signers). In a study by the same authors (Van Dijk et al., 2013b) on haptic spatial orientation abilities, it was the deaf group that outperformed hearing signers and non-signers indicating that for this type of processing, perceptual abilities change as a function of sensory deprivation.

Moreover, studies have revealed modality specific differences for changes in performance. Contrary to enhancements in visual detection tasks for congenitally and early deaf individuals, Heimler and Pavani (2014) did not find any behavioral differences to hearing controls in similar tasks in the tactile modality. Notably, none of their participants acquired a signed language from birth or in early childhood. Frenzel et al. (2012) report worse performance in tactile sensitivity in a young group of deaf participants. However, the lack of information on the participants’ background impedes a further interpretation of the results.

However, sometimes, findings from studies with different stimulus modalities are consistent. Pavani and Bottari (2012) point to an advantage of deaf compared to hearing individuals for visual detection, but discuss how in the visual modality, discrimination tasks tend to result in comparable outcomes in deaf and hearing groups instead. While Levänen and Hamdorf (2001) reported behavioral enhancements of congenitally deaf signers compared to hearing controls in the detection of tactile frequency changes, no group differences were observed in a tactile frequency discrimination task. This is in accordance with findings from a haptic spatial discrimination task in a study by Bolognini et al. (2012). Regarding the impact of neuroplasticity on the development of different perceptual functions, Cardin et al. (2020) argue that functional preservation and change must not rule each other out—instead, they might be based on different and yet simultaneously existing neural mechanisms (see also Land et al., 2016).

An interesting case of somatosensory processing in deaf and deafblind individuals are temporal tasks. Several studies have pointed to neural differences regarding spatial vs. temporal processing and the assumption that compared to hearing individuals, deaf individuals might show disadvantages in temporal processing (e.g., Heming and Brown, 2005; Bolognini et al., 2010, 2012; Papagno et al., 2016; Zimmermann et al., 2021).

Bolognini et al. (2012) found a correlation between auditory cortex involvement latency and behavioral performance in a temporal task. Later recruitment of auditory areas (as observed in the hearing control group) was associated with better behavioral outcomes. Following the *perceptual deficiency theory,* the authors argue that for the typical development of temporal processing skills, early auditory experience is needed. From this point of view, the earlier involvement of the STG for both tasks in the deaf group, as opposed to the later and specific activation for the temporal task in hearing individuals, would indicate a lack of crossmodal compensation as a result of deafness (see Scurry et al., 2020b, for fMRI results on lower proportions of directionally tuned voxels in primary somatosensory cortex in deaf compared to hearing individuals).

Other studies have reported similar behavioral outcomes for deaf and hearing groups in temporal processing (Bross and Sauerwein, 1980; Poizner and Tallal, 1987; Nava et al., 2008; Moallem et al., 2010). Identifying the critical factors for these differences in behavioral outcomes poses a challenge. Regarding their sensory experiences, the included samples of deaf individuals appear to be rather homogenous—all of them reported a congenital or early onset of deafness. Importantly, though, their linguistic experiences are not always available in full detail. For example, Heming and Brown (2005) describe that all deaf participants reported ASL as their first language—however, no further information on their actual age of acquisition is provided. Bolognini et al. (2012) included nine congenitally deaf participants in their temporal task. While two of them did not know a signed language, the other seven had acquired LIS before the age of 3 years. However, some of them grew up in hearing families and learned LIS in school, and it is not traceable what their daily linguistic experience in school and at home looked like.

The arguments by Bolognini et al. (2012) concerning a decrease in temporal perception skills as a function of auditory deprivation are supported by findings by Papagno et al. (2016). Here, congenitally deaf and late deafblind individuals both displayed better performance in spatial, but lower performance in temporal discrimination than the controls. However, no congenitally deafblind individuals were included—it could be speculated that their somatosensory perception is more enhanced than in sighted and late blind deaf individuals. Comparing congenitally and late deafblind individuals in the same experimental design would allow for an investigation of the impact of bisensory deprivation from birth versus the loss of a second sensory system later in life. Moreover, none of the deaf participants in the study had acquired a signed language from birth, and only two learned one later in life.

Sharp et al. (2018) showed higher error rates in crossed vs. uncrossed arms conditions in a tactile TOJ task. They suggest that this is due to difficulties in integrating the conflicting visual and somatosensory information in the deaf group. While the participants were congenitally deaf, only one out of 13 participants was a user of a signed language. Thus, it might be possible that the poorer performance of deaf participants in temporal tasks such as the TOJ conducted by Sharp et al. (2018) is due to a delay in first language acquisition and its impact on other cognitive functions. In deaf signers, one might expect an increased ability to navigate the crossed arms condition instead.

Space is a critical factor in sign language production and perception (Mathur and Rathmann, 2012; Shield and Meier, 2018). Deaf (and hearing) signers display advantages in spatial processing, such as mental rotation skills (Emmorey et al., 1993; Kubicek and Quandt, 2021). Because signers do not get visual feedback from their own language production, they rely more on somatosensory feedback and proprioception compared to speech production (Emmorey et al., 2009). For deafblind language users, tactile sign languages and tactile communication systems are perceived and produced through the somatosensory modality by the conveyer and the receiver (Edwards and Brentari, 2020, 2021). Papagno et al. (2016) argue that “discriminative touch is not so relevant in humans, while social touch is” Thus, for individuals who have acquired a visual-gestural or tactile signed language within the significant time windows of brain development, a significantly decreased performance in tactile processing would be unexpected—in particular for spatial, but also temporal tasks. Yet, some previous studies have pointed to opposite findings (e.g., Bolognini et al. 2012). It has been argued that when compared to vision and audition, touch is often underestimated regarding its information content (Gallace and Spence, 2014). Unlike the visual modality, the tactile modality has a high temporal resolution as well, and thus, the somatosensory system should provide reliable temporal information to deaf and deafblind individuals. Therefore, to explain the results in temporal tasks, it is crucial to address the role of language modality and acquisition on behavioral differences and neural changes in deaf and deafblind individuals compared to control groups. To date, especially, but not only the impact of acquiring and using a tactile sign language on other somatosensory perceptual functions remains inconclusive (Edwards, 2018). Clearly, more studies are needed to address this gap in the literature.

In tasks on tactile memory skills, the outcomes of several studies did not reveal a consistent pattern, either. Arnold and Heiron (2002) observed a faster completion time in deafblind compared to hearing participants, but similar accuracy outcomes. Notably, the etiologies and language backgrounds of the included deafblind individuals is not explained in detail. Papagno et al. (2017) did not find response time differences between deaf, blind, deafblind, and control participants, but the deaf and blind groups outperformed the controls in all other behavioral measurements. For the blind and deafblind groups, performance and age of Braille acquisition were negatively correlated, indicating Braille experience as an impacting factor for tactile short-term memory skills. Task performance was not correlated with onset of deafness (in deaf and deafblind participants) and blindness (in blind and deafblind participants). No correlation was found including LISt acquisition, however, only eight out of the 15 participants had learned LISt, and no information on their early language experiences is available. None of the deaf individuals acquired a signed language from birth, some never did. Thus, it could be speculated that at least some participants did experience a delayed acquisition of a first language. In a single-case study including a congenitally deafblind woman, Janssen et al. (2007) reported similar response time and more errors in tests compared to hearing controls in a tactile memory task.

For deaf participants, alterations in performance and neural responses have been found for multisensory processing, indicating neuroplasticity as a result of auditory deprivation (e.g., Karns et al., 2012; Hauthal et al., 2015; Villwock et al., 2022). Different behavioral outcomes in deaf compared to hearing groups have been demonstrated for static (e.g., Karns et al. 2012) and dynamic visuo-tactile stimulation (Villwock et al., 2022). Regarding neural responses in somatosensory processing, several studies have indicated signs of intramodal and crossmodal plasticity as a function of deafness (e.g., Levänen et al., 1998; Auer et al., 2007; Bolognini et al., 2012; Karns et al., 2012; Villwock et al., 2022; but see Hickok et al., 1997). For simple static stimulation, these patterns were observed in participants with different language backgrounds (Levänen et al., 1998; Auer et al., 2007). In a task including congruent and incongruent motion stimuli, Villwock et al. (2022) observed a more anterior distribution of the electrophysiological response as well as differences in the latency and the lateralization of a motion congruency effect. Because the participants were all congenitally deaf and acquired a signed language from birth, it cannot be concluded whether these differences are based on the experience of deafness, sign language usage, or both.

For deafblind individuals, two studies examining the neural response to tactile language input (Braille, Print on Palm, and hASL) revealed enhanced activation of language areas in a late deafblind (MEG study; Osaki et al., 2004), and in a congenitally deafblind and a sighted hearing participant (fMRI study; Obretenova et al., 2010). Moreover, Obretenova et al. (2010) observed increased bilateral activation in occipital cortex as well as enhanced occipital-temporal connectivity in the deafblind participant, but not the hearing user of hASL. This points to changes as a result of crossmodal plasticity, which is in accordance with findings of enhanced activation in auditory areas following tactile stimulation in deaf (e.g., Levänen et al., 1998; Karns et al., 2012), and in visual areas as a response to auditory stimulation in blind individuals (e.g., Bedny et al., 2011).

Taken together, the pattern is not clear, and sometimes, different outcomes were observed in very similar tasks. These inconsistencies may be due to a high variance regarding samples of deaf and deafblind participants and their sensory as well as linguistic experiences (Bavelier and Neville, 2002; Dye and Hauser, 2014). To shed light on sensory processing after auditory and audio-visual deprivation, future studies need to thoroughly distinguish between possible influencing factors. In some studies, participants were congenitally deaf or deafblind, whereas, in others, they had become deaf, blind, or deafblind later in life. Some acquired a language from birth, and these languages included, *inter alia*, (tactile) signed languages, spoken languages, and Braille. Some participants experienced a delayed acquisition of a first language. Some used a signed system based on the grammar of a spoken language (such as signed exact English, SEE), and some were non-signers. Keeping the diversity of the included samples in mind is crucial to identifying the deciding factors for possible differences in the neural response and behavioral outcomes.

In some cases, findings might have been misinterpreted due to a lack of information about the participants’ individual backgrounds. This could include sensory as well as linguistic experiences. A perspicuous example regarding language experience comes from previous work on selective attention in deaf children (for a review, see Dye and Bavelier, 2010). Several studies seemed to support the view that deafness negatively impacted attentional skills in children (e.g., Quittner et al., 1994; Smith et al., 1998). However, when the samples of children were controlled for language background, the results turned out differently. For example, in a visual–spatial attention task, a similar performance was observed in hearing children and deaf children who had learned a language from birth—in this case, ASL (Dye et al., 2009). Therefore, the individual language experiences of participants must be considered.

Importantly, only a minority of deaf children are born into deaf, signing families (approx. 5%, see Mitchell and Karchmer, 2004). They tend to grow up with a signed language, experience a typical language acquisition from birth, display fewer comorbidities, and have a smaller probability of undergoing neurological trauma (Dye and Bavelier, 2013; Lillo-Martin and Henner, 2021). On the other end of the language acquisition continuum, there are individuals who might never experience full access to a language. Deaf children born into hearing, non-signing families can be at risk of experiencing delayed first language exposure and atypical social communication (Dye and Bavelier, 2013; Wilkinson and Morford, 2020). Language deprivation has consequences on emotional, linguistic, and cognitive development (Mayberry et al., 2002; Morford, 2003; Humphries et al., 2012). Moreover, without input, some neural networks associated with language processing cannot typically develop (Mayberry et al., 2011). For example, the usual dominance of the left hemisphere as observed in deaf individuals acquiring a signed language early in life does not seem to occur in very late learners of a first language (Ferjan Ramirez et al., 2014). These findings point to a change in neural circuits involved in language processing after severe language deprivation. While the risk of delayed exposure to a first language is still a highly relevant issue for deaf children today (Wilkinson and Morford, 2020; Villwock et al., 2021), the situation is even more alarming for children who are born deafblind (Edwards and Brentari, 2021). Therefore, participants from these groups will display highly variable linguistic backgrounds.

Most experimental studies with deaf and deafblind individuals have followed a purely quantitative approach for collecting participants’ information, using questionnaires and surveys with often rather limited content. However, especially for deafblind participants, even a very thorough quantitative approach may not be sufficient to fully capture an individual’s experience. Instead, a deeper qualitative investigation on the single-case level would be needed. This points to two important considerations: First, to draw general clear conclusions, the included samples need to be as homogenous as possible concerning their sensory experiences and etiologies, and different groups of participants performing the same task should be included in studies. Second, the individual language backgrounds of the participants must be taken into account. For example, testing somatosensory processing in deaf L1M1 signers might not be enough to understand how deaf individuals process input from the environment. Furthermore, too often, a deficit-oriented point of view might have resulted in a lack of studies on, for example, tactile languages (Henner 2022).

### Conclusion

To conclude, when conducting studies on the processing of touch with deaf and deafblind individuals, a thorough investigation of individual experiences is crucial for explaining the results. Including such measures could shed light on the reasons for possible changes concerning the remaining sensory modalities—that is, for neuroplasticity. Importantly, the participants’ etiologies, as well as their language backgrounds, need to be considered in more detail. This review of the current research on basic perceptual functions in deaf and deafblind individuals focused on behavioral outcomes and crossmodal plasticity. It demonstrates that neither sensory nor linguistic backgrounds alone provide sufficient knowledge about an individual’s experience. To date, the results do not provide a clear picture, and sometimes, findings from different studies with rather similar tasks show conflicting information. Hence, the impact of deafness and deafblindness on the processing of touch remains not well understood. Given the highly variable language backgrounds in deaf and deafblind communities, examining individual experiences is crucial in order to understand the development of the somatosensory system. For example, delayed access to a first language, and even more so a serious language deprivation may have an impact on other, basic perceptual functions. Ideally, studies should aim to include clearly defined groups of participants and apply similar tasks to samples with different sensory and linguistic experiences. Including a broad range of participants is demanding, but important to identify the deciding factors for possible differences in the neural response and behavioral outcomes. This comprehensive perspective can be considered to strategically disentangle the impact of sensory experience (deprivation) and language experience on basic sensory processing—and vice versa. By providing novel information on the connection between perceptual functions and individual experience, it can contribute to a better understanding of the human brain and its plasticity.

## Author contributions

AV wrote the first draft of the manuscript. KG and AV wrote sections of the manuscript. All authors contributed to manuscript revision, read, and approved the submitted version.

## Funding

The article processing charge was funded by the Deutsche Forschungsgemeinschaft (DFG, German Research Foundation)—491192747 and the Open Access Publication Fund of Humboldt-Universität zu Berlin.

## Conflict of interest

The authors declare that the research was conducted in the absence of any commercial or financial relationships that could be construed as a potential conflict of interest.

## Publisher’s note

All claims expressed in this article are solely those of the authors and do not necessarily represent those of their affiliated organizations, or those of the publisher, the editors and the reviewers. Any product that may be evaluated in this article, or claim that may be made by its manufacturer, is not guaranteed or endorsed by the publisher.
